# Supplementary material for: The Sequence Dependent Nanoscale Structure of CENP-A Nucleosomes
Source: Int J Mol Sci. 2022 Sep 27;23(19):11385. doi: 10.3390/ijms231911385 (PMC9569828; doi:10.3390/ijms231911385)
Supplement: Supplementary file 1 [file ijms-23-11385-s001.zip › ijms-1910808-supplementary.pdf]

Supplementary Information for

Sequence Dependent Nanoscale Structure of CENP-A Nucleosomes

Tommy Stormberg[a] and Yuri L. Lyubchenko[1,*]
[1]Department of Pharmaceutical Sciences, University of Nebraska Medical Center, Omaha, NE 68198-6025.

***Corresponding author:** Yuri L. Lyubchenko, 402-559-1971, Nebraska Medical Center, Omaha, NE 68198-6025
Email: ylyubchenko@unmc.edu

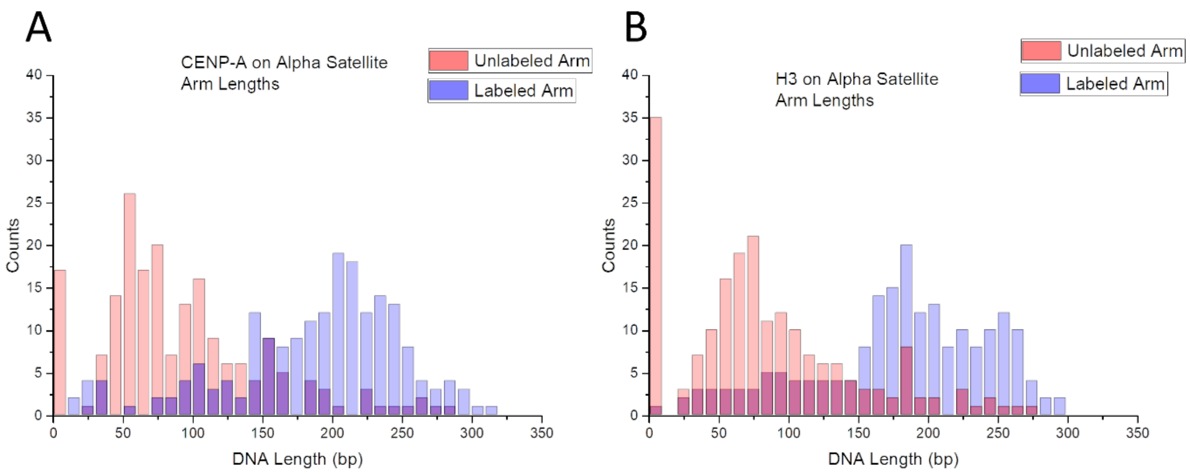

Fig. S1. Free DNA flank length measurements for nucleosomes assembled on the α-satellite substrate.

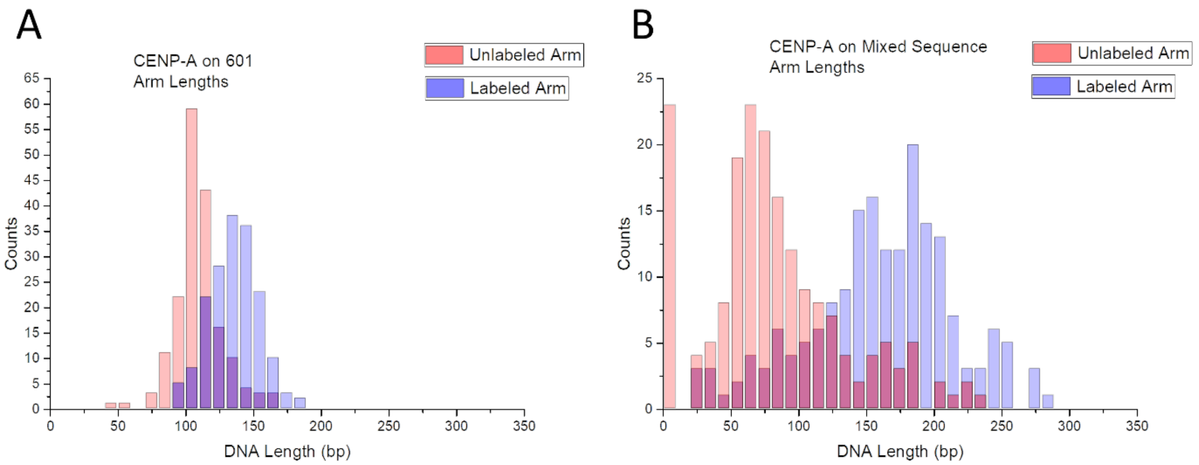

Fig. S2. Free DNA flank measurements for CENP-A nucleosomes assembled on the 601 motif **(A)** and the non-specific DNA **(B)**.

Alpha-Satellite Sequence:
5'GATGTGCTGCAAGGCGATTAAGTTGGGTAACGCCAGGGTTTTCCCAGTCACGACGTTGTAAAACG
ACGGCCAGTGAATTCGAGCTCGGTACCTCGCGAATGCATCTAGATGACCATTGGATTGAACTAACAG
AGCTGAACACTCCTTTAGATGGAGCAGATTCCAAACACACTTTCTGTAGAATCTGCAAGTGGATATTT
GGACTTCTCTGAGGATTTCGTTGGAAACGGGATAAAATTCCCAGAACTACACGGAAGCATTCTCAGA
AACTTCTTTGTGATGAAGGGCGAATTCGAATCGGATCCCGGGCCCGTCGACTGCAGAGGCCTGCAT
GCAAGCTTGGCGTAATCATGGTCATAGCTGTTTCCTGTGTGAAATTGTTATCCGCTCACAATTCCACA
CAACATACG3'

601 Sequence:
5'GATGTGCTGCAAGGCGATTAAGTTGGGTAACGCCAGGGTTTTCCCAGTCACGACGTTGTAAAACG
ACGGCCAGTGAATTCGAGCTCGGTACCTCGCGAATGCATCTAGATGACACAGGATGTATATATCTGA
CACGTGCCTGGAGACTAGGGAGTAATCCCCTTGGCGGTTAAAACGCGGGGGACAGCGCGTACGTG
CGTTTAAGCGGTGCTAGAGCTGTCTACGACCAATTGAGCGGCCTCGGCACCGGGATTCTCCAGGTC
ATCGGATCCCGGGCCCGTCGACTGCAGAGGCCTGCATGCAAGCTTGGCGTAATCATGGTCATAGCT
GTTTCCTGTGTGAAATTGTTATCCGCTCACAATTCCACACAACATACG3'

Non-Specific Sequence:
5'GATGTGCTGCAAGGCGATTAAGTTGGGTAACGCCAGGGTTTTCCCAGTCACGACGTTGTAAAACG
ACGGCCAGTGAATTCGAGCTCGGTACCTCGCGAATGCATCTAGATGACCCTGGGGTGCCTAATGAG
TGAGCTAACTCACATTAATTGCGTTGCGCTCACTGCCCGCTTTCCAGTCGGGAAACCTGTCGTGCCA
GCTGCATTAATGAATCGGCCAACGCGCGGGGAGAGGCGGTTTGCGTATTGGGCGCTCTTCCGGACA
TCGGATCCCGGGCCCGTCGACTGCAGAGGCCTGCATGCAAGCTTGGCGTAATCATGGTCATAGCTG
TTTCCTGTGTGAAATTGTTATCCGCTCACAATTCCACACAACATACG3'

Fig. S3. Sequences used for DNA substrates.
